# A study to determine a capillary alternative to the gold standard oral glucose tolerance test - *Protocol*

**DOI:** 10.12688/wellcomeopenres.23028.2

**Published:** 2026-01-29

**Authors:** Rabbi Swaby, Claire Scudder, Tabitha Randell, M. Loredana Marcovecchio, Kathleen Gillespie, Yuk-Fun Liu, John A Todd, Gareth Dunseath, Steve Luzio, Colin Dayan, Rachel E J Besser

**Affiliations:** 1 Diabetes and Inflammation Laboratory, Centre for Human Genetics, Nuffield Department of Medicine, Bukhman Centre for Research Excellence in Type 1 Diabetes,NIHR Oxford Biomedical Research Centre, University of Oxford, Oxford, England, UK; 2 Nottingham Children's Hospital, Nottingham University Hospitals NHS Trust, Nottingham, England, UK; 3 Department of Paediatrics, University of Cambridge, Cambridge, UK; 4 Diabetes and Metabolism Unit, University of Bristol Translational Health Sciences, Bristol, England, UK; 5 School of Life Course Sciences, King's College London, London, UK; 6 Diabetes Department, Guy's and St Thomas' NHS Foundation Trust, London, UK; 7 College of Medicine, Swansea University, Swansea, UK; 8 Clinical Diabetes and Metabolism, Cardiff University School of Medicine, Cardiff, Wales, UK; 9 University of Oxford Department of Paediatrics, Oxford, England, UK

**Keywords:** Capillary, glucose, oral glucose tolerance test, method comparison, feasibility, acceptability, type 1 diabetes, monitoring, follow-up, screening

## Abstract

Type 1 diabetes (T1D) is a chronic condition caused by the immune destruction of the pancreatic beta cells. T1D has recognised asymptomatic pre-clinical stages, providing an opportunity for early diagnosis, education and treatment which may delay the onset of symptoms. The oral glucose tolerance test (OGTT) is the gold standard method to stage and monitor early-stage T1D, which can be poorly tolerated and may contribute to marked loss to follow-up.

Our study aims to test the accuracy, feasibility, and acceptability of a capillary alternative (‘GTT@home’ test kit) to the gold standard OGTT.

We will invite 45 children and young people (CYP) across the spectrum of glycaemia with or without diabetes, from established research platforms or clinical care, to have a standard 2-hour OGTT, with capillary samples collected alongside their venous samples, at 0 and 120 minutes. A subgroup (n=20) will also have 60-minute capillary and venous samples collected.

We will also invite 45 CYP from established research platforms, who are known to have two or more islet autoantibodies and are not on insulin, to undergo a capillary OGTT at home, using the GTT@home kit.

We will assess the agreement of capillary and venous glucose and measure diagnostic accuracy by calculating the sensitivity and specificity of capillary measures at established diagnostic thresholds (fasting [5.6 mmol/L, 7.0 mmol/L], 60 minutes post glucose load [11.1 mmol/L] and 120 minutes post glucose load [7.8 mmol/L and 11.1 mmol/L]), using venous glucose as the gold standard.

These studies will inform our understanding of whether the GTT@home device can be used in CYP in routine clinical care.

## 1. Plain Language Summary

Type 1 diabetes (T1D) is a chronic condition caused by the immune system damaging the pancreas, the organ which makes insulin, and impacts every aspect of a person’s life. T1D has recognised stages before symptoms develop, allowing early diagnosis, education and treatment which may delay the onset of symptoms. Early diagnosis often relies on a test called the oral glucose tolerance test (OGTT). This test usually involves an individual fasting for 8 hours overnight and then having blood samples taken to measure blood glucose (sugar) levels before drinking a sugary drink, and over the following 2 hours. It is commonly used but not well tolerated, possibly because it requires a drip to be inserted into a vein, is time-consuming and requires travel to a healthcare setting.

Our study aims to test whether we can do an OGTT using a finger-prick to test glucose, at home, using the ‘GTT@home’ test. The finger-prick creates a drop of blood, which is done before and two hours after drinking a sugary drink. The test kit has a built-in timer and can be scanned using a smartphone to send the results securely to a clinician to be reviewed.

We plan to invite 90 children and young people, across two groups to assess the GTT@home.

Group 1 will assess the accuracy of measuring glucose from a finger-prick blood test, compared to a blood test from the vein, at 0 and 120 minutes. A subgroup will be invited to have an additional sample taken at the 60-minute time point.

Group 2 will assess how well the GTT@home test works when done at home and how acceptable it is. This will only be offered to those known to be at risk of T1D.

These studies will help us understand whether the GTT@home can be used in routine care.

## 2. Introduction

### 2.1. Type 1 diabetes is an important health condition

Type 1 diabetes (T1D) is a chronic autoimmune condition, with an annual incidence of 31 per 100,000 in the general population, affecting 1 in 490 children and young people (CYP) under 15 years of age in the UK ^1^. T1D occurs as a result of genetic and environmental factors, leading to islet-cell autoimmunity and T-cell mediated pancreatic beta-cell destruction. This process can take months or years to develop and leads to dysglycaemia and eventually, hyperglycaemia with osmotic symptoms. Diabetic ketoacidosis is present in ~38% of those newly diagnosed with T1D present. This is a decompensated metabolic state often requiring intensive hospital management ^2, 3^.

### 2.2. Stages and classification of Type 1 diabetes

T1D now has well-described stages, defined by the gold standard oral glucose tolerance test (OGTT) ( Table 1). Stages 1-2 precede clinical disease requiring insulin treatment (stage 3). These stages can also be used to counsel families about risk of progression to clinical disease ^4, 5^.

**Table 1.** Staging of T1D using OGTT or HbA1c criteria ^4, 26^.

Staging	Plasma glucose or HbA1c level	Interpretation
Stage 1	**Fasting glucose** < 5.6 mmol/L (< 100 mg/dL) *or* **Two-hour glucose** < 7.8 mmol/L (< 140 mg/dL)	Normoglycaemia
Stage 2	**Fasting glucose** 5.6 – 6.9 mmol/L (100 – 125 mg/dL) *or* **Two-hour glucose** 7.8 – 11.0 mmol/L (140 – 199mg/dL) ^*^ *or* **HbA1c** 39 – 47 mmol/mol (5.7 – 6.4%) or ≥ 10% increase in HbA1c	Dysglycaemia
Stage 3	**Fasting glucose** ≥ 7.0 mmol/L (≥ 126 mg/dL) *or* **Two-hour glucose** ≥ 11.1 mmol/L (≥ 200 mg/dL) ^*^ *or* **HbA1c** ≥ 48 mmol/mol (≥ 6.5%)	Clinical T1D

* a blood glucose of ≥11.1 mmol/L at any intermediate point (30, 60, or 90-minute) in an OGTT is interpreted as impaired glucose tolerance (or dysglycaemia).

Stage 1 is defined by the presence of two or more islet autoantibodies (IAb) with normoglycaemia and stage 2 is the development of dysglycaemia ^6^. The presence of one IAb poses a 10–15% risk of developing stage 3 T1D by the age of 18, whilst two or more presents an 80–90% risk by age 18, with a lifetime risk approaching 100% ^7, 8^. This latency period offers an opportunity for those identified to be educated and monitored, reducing the risk of presentation in DKA and allowing a controlled introduction to insulin therapy ^4^.

### 2.3. Screening can identify children before clinical symptoms

Several population studies have demonstrated possible benefits of identifying children during the pre-clinical phase of T1D (stage 1 and 2). These benefits include a reduction in the rate of DKA at presentation (by ~90%), lower hospitalisation rates and lower HbA1c at diagnosis ^9– 12^. In Germany the Fr1da study screened approximately 170,000 children aged 2–5 years by measuring capillary IAb during “well child” checks in primary care; in the US the Autoimmunity Screening for Kids (ASK) study opportunistically screened children aged 1–17 years for IAb and coeliac antibodies; The Environmental Determinants of Diabetes in the Young (TEDDY) group screened infants for high-risk T1D genetics across Europe and the United States. Identification during the pre-clinical phase also gives the opportunity for treatment with drugs to delay T1D onset, one of which (teplizumab an anti-CD3 monoclonal antibody) has already received licencing by the Food and Drug Administration in the US and is under regulatory assessment in Europe and the UK ^13, 14^.

### 2.4. Follow-up in children who are in early stage T1D

Several methods can be used to assess disease progression and predict future risk of T1D, with the OGTT being the gold standard for both staging and monitoring progression.

### 2.5. Oral glucose tolerance test

The OGTT involves ingestion of a standard glucose load (1.75 g/kg, 75g maximum) and blood sampling before, during and 2 hours after glucose ingestion. This requires cannulation, is time-consuming and requires travel to have this undertaken in a healthcare setting. This may contribute to poor adherence when used as part of follow-up, with loss to follow-up rates in research studies reaching as high as ~50% ^15, 16^. However, the OGTT does provide valuable data which allows the staging of T1D ( Table 1) and informs the risk of progression to clinical disease. Combining data from the OGTT with other metrics allows the calculation of risk progression scores, such as the five-timepoint Diabetes Prevention Trial-Type 1 Risk Score (DPTRS), DPTRS60, Index60, M120 and Progression Likelihood score ^17^, (see Table 2). These risk progression scores achieve improved area under the receiver operating characteristics curve compared to impaired glucose tolerance alone in predicting progression to clinical diabetes (stage 3) ^18– 22^.

**Table 2.** Information on T1D risk progression scores ^18– 22^.

Risk Progression Score	Number of OGTT Timepoints	Required Metrics
DPTRS	5	Age, BMI, plasma glucose and C-peptide at 0, 30, 60, 90, 120 minutes
DPTRS60	2	Age, BMI, fasting C-peptide and 60-minute plasma glucose and C-peptide
Index60	2	Fasting C-peptide, 60-minute plasma glucose and C-peptide
M120	1	Age, sex, BMI, HbA1c, insulinoma antigen-2 status, C-peptide, and plasma glucose at 120 minutes.
Progression Likelihood score	1	HbA1c, 90-minute plasma glucose, islet-antigen presence and titre.

### 2.6. The OGTT is an imperfect gold standard test

Despite its status as the gold standard test for staging and calculation of T1D risk progression, several factors can impact the reliability of the OGTT. These have been categorised as pre-analytical, analytical and post-analytical factors ^23^. Pre-analytical factors, such as patient preparation, sample handling and patient physiology account for around 55% of reproducibility issues ^24^. When glucose samples are not handled as per standard conditions (plasma separated and stored at -20˚C or colder within 30 minutes), it can lead to an increased false negative rate, as 5–7% per hour of whole blood glucose is metabolised in the test tube ^23, 25^.

### 2.7. Other markers of dysglycaemia

There are other less invasive measures that can be useful in monitoring glucose levels in early-stage T1D but these are inferior to the gold standard OGTT, and are summarised below ( Table 3).

**Table 3.** Monitoring tools available for children with pre-clinical T1D, adapted from ISPAD guidelines ^4^.

Monitoring tool	Pros	Cons	Information gained from test
OGTT	Gold standard Ability to stage and monitor disease	Invasive Time-consuming Requires multiple blood draws over 2 hours	Metabolic staging Risk of progression and can be combined with other metrics to inform risk scores for disease progression calculation (DPTRS, DPTRS60, Index60, M120, PLS)
HbA1c	Highly specific Option for capillary sample Ability to stage and monitor disease	Insensitive Affected by other disease states	Risk of progression to stage 3 T1D: HbA1c >5.7% or 10% rise over 3–12 months
CGM	Can be used at home	Cost Access to technology Optimal duration of monitoring unknown Can’t be used to stage disease Acceptability uncertain in the general population	Risk of progression to stage 3 T1D over 1 year: 10% time >7.8mmol/L (140mg/dL) and over 2 years: 5% time >7.8mmol/L Real-time monitoring
Random venous glucose	Cheap	Requires blood test Insufficient to stage disease	Similar to the 2-hour post OGTT value
Self-monitored blood glucose	Simple Can be done at home	Timing and frequency of testing unknown	Immediate result given

### 2.8. Proposed alternative test to the standard OGTT: the capillary OGTT

***2.8.1. Description of the capillary OGTT method***

The capillary OGTT device (GTT@home) is a novel potential alternative to the standard OGTT which aims to overcome some of the issues seen with the standard OGTT.

It provides step-by-step instructions on how to perform the OGTT at home without training, collects the capillary blood samples and performs glucose tests at two time points (0 and 120 minutes). It is calibrated to convert capillary to venous plasma glucose results, using 0.6 microlitres of capillary blood. As the sample is processed immediately on the device, it reduces the burden of sample processing and potentially avoids the need for the participant to attend a healthcare setting for sample processing.

The device is manufactured by Digostics Ltd and uses two glucose dehydrogenase sensors to take the capillary glucose readings. The enzymatic reaction creates an electrochemical signal which transfers the data to the detachable data record, which also incorporates the wireless data transfer used in smartphones (near-field communication). It has a built-in 2-hour timer and uses audible alarms to inform the participant when to take the capillary sample. Once complete, the raw data can be uploaded to a cloud-based server for analysis via a smartphone app. Alternatively, the detachable data record can be posted to the study team.

***2.8.2. Published literature using the GTT@home device***

The GTT@home device has a UKCA/CE mark (UK Conformity Assessed, Conformité Européene, or European Conformity marking) to perform an OGTT at home, to aid in the diagnosis of diabetes in adults, and in children under adult supervision. It has been evaluated in multiple adult studies, including one involving 100 women with and without glucose intolerance, which found excellent agreement between the device and laboratory glucose values ^27^. A similar study involving adults with and without type 2 diabetes also demonstrated good agreement between capillary and laboratory glucose measures using the device, with most users reporting that it was easy to use ^28^. This accuracy was demonstrated in populations whose average haematocrit fell within normal limits, with bias shown when haematocrit levels were high or low ^29, 30^. There has been no published validation of this device or methodology in children, nor individuals with early-stage T1D. This study would aim to provide evidence of its reliability, feasibility, and acceptability in children, as an alternative to the standard venous OGTT.

## 3. Study objectives

**Overall aim:** To determine the reliability, feasibility, and acceptability of the capillary OGTT method as an alternative to the standard venous OGTT in children with early-stage T1D ( Table 4).

**Table 4.** Study objectives.

COHORT 1		
Objectives	Analyses	Timepoint(s) of evaluation of this outcome measure (if applicable)
**Primary Objective** 1. To determine the agreement of capillary blood glucose to venous blood glucose levels during a standard OGTT	1. Agreement between capillary and blood glucose measures.	1. Baseline (fasting) time = 0 minutes 2. 60 minutes post glucose load (subgroup only) 3. 120 minutes post glucose load.
**Secondary Objectives** 1. To determine the diagnostic accuracy of capillary blood glucose levels at diagnostic thresholds	1. Sensitivity and specificity of capillary glucose at 5.6mmol/L, 7.0mmol/L (fasting), 11.1 mmol/L (60 min), and 7.8mmol/L and 11.1mmol/L (120 min).	End of study
2. To assess the acceptability of the capillary OGTT device	1. Acceptability by questionnaire (Parent +/- child)	End of study
COHORT 2		
**Objectives**	**Outcome Measures**	**Timepoint(s) of evaluation** **of this outcome measure (if** **applicable)**
**Primary Objective** 1. To assess the feasibility of using the capillary OGTT device in the home environment	1. Proportion of successful glucose readings at 0, 120 minutes 2. Proportion of errors/missing glucose readings 3. Proportion of adverse events	1. Baseline (fasting) time = 0 minutes 2. 120 minutes post glucose load 3. End of study
**Secondary Objectives** 1. To assess the acceptability of the capillary OGTT device when used at home	1. Acceptability by questionnaire (Parent +/- child)	End of study

## 4. Methods

The protocol for this observational study was written following The Strengthening the Reporting of Observational Studies in Epidemiology (STROBE) Statement ^31^.

### 4.1. Study design

***4.1.1. Cohort 1 (Simultaneous venous and capillary OGTT)***

We aim to assess the ability of the capillary OGTT to be a reliable and acceptable alternative to the standard venous OGTT in children ( Table 5).

**Table 5.** Inclusion and exclusion criteria.

Inclusion Criteria		
Cohort 1
1) Willing and able to give informed consent for participation, or assent with parental consent 2) Aged < 18 years old 3) Able to consume oral glucose drink within 10 minutes 4) Undergoing an OGTT, or consent to have one
Cohort 2
1) Confirmed known ≥ 2 IAb antibodies 2) Willing and able to give informed consent for participation, or assent with parental consent 3) Aged < 18 years old 4) Able to consume oral glucose drink within 10 minutes
Exclusion Criteria
Cohort 1
1) Any known haemoglobinopathy 2) Cystic fibrosis-related diabetes 3 Non-English speaker
Cohort 2
1) Any known haemoglobinopathy 2) Known clinical diabetes and on treatment 3) Non-English speaker 4) No recent weight available (within 3 months of study visit) and unable to obtain new weight measurement

This study will be undertaken within UK clinical and research settings, led by either a healthcare professional or a research nurse. These settings will include NHS hospitals and research facilities.

Children undergoing a standard venous OGTT will be invited to complete a simultaneous capillary OGTT. Glucose samples will be collected at 0 and 120 minutes, and for 20 participants, an additional 60-minute sample will be collected. A full blood count (FBC) will also be collected to measure haematocrit, which is known to affect the accuracy of capillary glucose sampling when significantly outside the normal range ( Table 6).

**Table 6.** Breakdown of samples required for the planned OGTT (clinical samples) and research samples.

Time (mins)	Clinical Samples ^1^	Research Samples		
	Venous glucose (2ml)	Venous glucose (2ml)	Capillary glucose (<1µL)	FBC (2ml)
0	X	X	X	X
60 *	(X)	(X)	(X)	
120	X	X	X	

1. Clinical samples may vary in number and timing depending on the setting the test is being performed. * The 60-minute blood glucose value will only be taken in the Oxford subgroup. ** For participants with T1D, they will not have clinical samples taken, just research samples.

Participants will be asked to complete an acceptability questionnaire immediately following the OGTT.

***4.1.2. Cohort 2 (Capillary OGTT at home)***

In this cohort (running concurrently alongside cohort 1) we aim to assess the acceptability and feasibility of a capillary OGTT device in children and young people with early-stage (known stage 1 or 2) T1D at home.

This will take place in the home of participants, with written and video instructions provided.

Children known to be positive for ≥ 2 IAb will be invited to take part and will be sent a capillary OGTT test kit which will include a glucose drink, lancets and instructions (written and video).

They will fast overnight (from midnight the night before, for a minimum of 8 hours) before completing a 2-hour OGTT using the test kit and instructions provided. Glucose samples will be collected at 0 and 120 minutes. Participants will be asked to complete a questionnaire following completion of the OGTT, to obtain information about acceptability.

### 4.2. Participants

***4.2.1. Cohort 1***

45 children < 18 years of age, will be invited to participate. The sources of these participants will include:

1. In-hospital clinical setting (OGTT being done for clinical reasons e.g., to diagnose diabetes)
2. Research setting (OGTT being done in a healthcare setting as part of a research study e.g., An Innovative Approach Towards Understanding and Arresting Type 1 Diabetes [INNODIA], Barts-Oxford [BOX] Family Study)
3. Children with clinical T1D (invited to undergo an OGTT for study purposes)

We aim to recruit CYP < 18 years across a range of ages and stages of T1D. To ensure we have a spread of glucose values; we will aim to recruit approximately equal numbers of participants with normoglycaemia, dysglycaemia and hyperglycaemia, according to the definitions above ( Table 1). To aid in recruitment stratification, we will use a recent HbA1c (within the previous 6 months), where available, to aid the categorisation of glycaemia. A HbA1c of < 5.7% (< 39mmol/mol) will be defined as normoglycaemia, a HbA1c of 5.7 – 6.4% (39 – 47mmol/mol) will be defined as dysglycaemia and a HbA1c ≥ 6.5% (≥ 48mmol/mol) will be defined as hyperglycaemia ^26^.

***4.2.2. Cohort 2***

45 CYP < 18 years of age, who are identified as having ≥ 2 IAb will be invited to participate. The sources of these participants will include:

1. Clinical care
2. Research setting (under follow-up in a research study e.g. INNODIA, BOX, TrialNet [an international network of academic institutions involved in the delivery of T1D research]) and who consented to be contacted about future research
3. Participants meeting eligibility criteria (i.e. ≥ 2 IAb) from cohort 1.

### 4.3. Recruitment

For participants identified through clinical care, clinical teams will approach parents and/or young people aged 16 – 18 years before their upcoming OGTT, and contact can be made in person, or via telephone or email. Local research team members will make the first contact with potential participants recruited through research platforms; contact will then be made by our research team if permission is given for this. Age-appropriate study information sheets will be given to those who agree to receive more information about the study.

Participants and parents will then be given at least 24 hours (or longer as required) to read the study information sheet, to allow them to make an informed decision about their participation.

## 5. Study procedures

### 5.1. Baseline assessments

***5.1.1. Cohort 1***

Baseline demographic data will be collected including age, gender, date of birth, height, weight, and ethnicity. Information on the reason for the OGTT, whether done as part of clinical care or research, recent HbA1c (within 6 months of expected study visit), and any known diagnoses will also be collected. Additional study data will be collected as described below.

***5.1.2. Cohort 2***

The research team will collect baseline demographic data including age, gender, date of birth and ethnicity via video call (Microsoft Teams ^32^) or telephone. The source of recruitment and diabetes stage (if known) will also be collected (this may also be obtained from the referrer). If the participant has been weighed by the referrer within 3 months of the study visit, that weight will be used for this study. If not, we will require the participant to be weighed at their local primary care setting. Additional study data will be collected as described below.

### 5.2. Subsequent visits

***5.2.1. Cohort 1***

Participants may be undergoing regular OGTTs (e.g. 6-monthly). This may result in being invited to take part for a second time. Any participants attending a second study visit will continue under their initial study ID (not re-enrolled) and acceptability questionnaires will not be repeated.

***5.2.2. Cohort 2***

Participants will only be required to attend one study visit. In the event of an abnormal result, they may require additional support, as outlined in Section 8.

### 5.3. Outline of study visit(s)

***5.3.1. Cohort 1***

This will take place in either a clinical or research setting. Study procedures will be carried out by a trained member of the clinical or research team.

Description of study procedure(s):

1. Participants advised to continue normal, balanced diet in the lead up to their OGTT (150g/day of carbohydrate intake for most children) ^33^.
2. Participants must fast from midnight (minimum 8 hours) before their test and may only have water.
3. Participants must avoid exercise and rest for the duration of the test.
4. Those with T1D will continue their long-acting insulin, omit their morning rapid-acting insulin and have their blood glucose checked. If the result is 4 – 11mmol/L they can proceed. If their blood glucose is >15 mmol/L they must check their ketones and treat appropriately, and not proceed.
5. Participant will attend a clinical or research setting as per usual process for their planned OGTT.
6. Demographic and baseline data will be taken and recorded on REDCap (see Section 5.1).
7. Participant will wash and dry their hands (with soap and water, not hand gel/sanitiser).
8. The clinician or researcher will insert an intravenous cannula.
9. After 10 minutes, a 2ml venous sample (fluoride oxalate) for plasma glucose measurement will be taken and a 2ml EDTA sample will be taken for haematocrit (via full blood count measurement). At the same time, a capillary sample for glucose measurement will be taken using a disposable safety lancet and the GTT@home kit.
10. The glucose drink will be consumed within 10 minutes (1.75g/kg or 75g max). For participants with known stage 3 T1D, reduce oral glucose dose (1g/kg or max 75g).
11. The timer will be started following the consumption of the glucose drink.
12. Participant will re-wash and dry their hands. A capillary blood sample will then be taken at 120 minutes, at the same time as a venous plasma glucose sample.
13. Once the test is complete, the participant/research nurse will scan the data record using their smartphone and Digostics app **and** follow step 14.
14. Participant to complete acceptability questionnaire.
15. Once the test is complete, the clinical team will remove the data record from the kit and store it in the case report form (CRF), along with the acceptability questionnaire.
16. All venous glucose samples will be processed (centrifugation, freezing ≥1ml of plasma [-20 to -80˚C] within 30 minutes.

***5.3.2. Cohort 1 (subgroup – will have a 60-minute sample collected)***

Participants recruited at the Oxford site will be invited to form the 60-minute subgroup. These participants have an additional 60-minute venous and capillary glucose sample taken. Otherwise, the study procedures will be as outlined in Section 5.3.1.

***5.3.3. Cohort 2***

Study procedures will be carried out by a parent of the participant at home. The capillary OGTT device along with a pre-made glucose drink (75g in 250ml glucose) will be sent to participants at home. They will be provided with written instructions/video provided by the manufacturer, on how to use the device and complete the OGTT. If the participant has been weighed within 3 months of the study visit by their referrer (research or clinical), that weight will be used. Otherwise, the participant will be required to be weighed at their local healthcare care setting.

1. Participants must fast from midnight before their test (minimum 8 hours) and may only have water.
2. Participants must avoid exercise and rest for the duration of the test.
3. Demographic and baseline data will be taken via video call and recorded on REDCap (see Section 5.1).
4. Participants will prepare the glucose load (1.75g/kg, max dose 75g) using a measurement device provided.
5. Participants will wash and dry their hands (with soap and water, not hand gel/sanitiser).
6. The participant/parent will use the provided lancet to produce a drop of capillary blood from the side of a finger.
7. Participant/parent will collect capillary glucose measurement with the drop of blood using the first glucose testing strip.
8. Participants will consume the glucose drink provided within 10 minutes.
9. Immediately after consuming the drink, the participant/parent will start the timer on the device.
10. When the timer sounds, indicating 120 minutes, they will stop the timer.
11. Participant will re-wash and dry their hands.
12. Participant/parent will use a new lancet to produce a drop of capillary blood from the side of a finger.
13. Participant/parent will collect a second capillary glucose measurement with a second drop of blood using the second glucose testing strip.
14. Once the test is complete, the participant/parent will scan the data record using their smartphone and Digostics app **and** follow step 15.
15. Once the test is complete, the participant/parent will detach the data record from the OGTT device. The acceptability questionnaire can be completed on REDCap or returned in the post, with the data record, to the research team at the Centre for Human Genetics, using the pre-paid envelope provided.

***5.3.4. Following sample collection***

**Cohort 1**

The clinician/researcher carrying out the test will ensure the participant is well before leaving the building.

**Cohort 2**

The study team will be contactable to support with any issues following the test.

***5.3.5. Acceptability assessment***

**Cohort 1**

The acceptability of the capillary OGTT device will be assessed using a questionnaire given immediately after the OGTT has finished and completed before departure.

***5.3.6. Cohort 2***

The acceptability of the capillary OGTT device, along with a record of any adverse events, will be assessed using a questionnaire completed after the OGTT, to be completed on REDCap or returned by post in a pre-paid envelope (identified by study number alone).

***5.3.7. Potential side effects using the GTT@home device***

No physical side effects from using the capillary OGTT device are anticipated. Slight pain or bruising may result from using the lancet (finger-prick device), which is used to provide the sample that the capillary OGTT device collects. To reduce discomfort, we will provide a smaller lancet (depth of approximately 1.5mm) for children under 8 years of age ^34^. The lancet is CE marked, as is the GTT@home test kit, which is also UK CA marked.

## 6. Data analysis and statistical plan

### 6.1. Determining the accuracy of the capillary OGTT device (GTT@home)

We will assess the strength of association over the whole range of diagnostic glucose values between the standard venous and capillary glucose measures by summary statistics reported for the glucose measurements (mean, range, standard deviation). A Bland-Altman plot will be used to assess the bias between the two methods, and the limits of agreement.

We will further assess the ability of capillary glucose levels to correctly classify five diagnostic glucose thresholds ( Table 1), of which two are fasting (5.6 mmol/L, 7.0 mmol/L), one is at 60 minutes (11.1 mmol/L) and two are stimulated 120-minute levels (7.8 mmol/L and 11.1 mmol/L), using receiver operating characteristic (ROC) curves, with corresponding specificities and sensitivities.

As haematocrit is known to influence capillary glucose measurement, we will also report summary statistics for haematocrit measurement (mean, range, standard deviation), via the measurement of full blood count.

### 6.2. Determining the acceptability of the capillary OGTT device (GTT@home)

We will assess the usability of the GTT@home using a traditional Likert scale (strongly disagree, disagree, neutral, agree, strongly agree) and a visual pain score (Wong-Baker Faces scale). For participants aged over 16 years, a questionnaire should be completed by the participant. For participants aged under 16 years, the questionnaires should be completed by both the participant and the adult guardian, as adapted from Liu *et al.*, 2017 ^35^.

### 6.3. Sample size calculation

***6.3.1. Cohort 1***

We aim to recruit 45 participants and will compare standard venous OGTT (fasting and 120 minutes) glucose with the capillary OGTT test. We will perform a subgroup analysis on our Oxford cohort to include glucose measurements at 0, 60 and 120 minutes. The recruitment target is justified based on the proposed feasibility of recruiting from existing research platforms or clinical care. We will undertake an interim analysis after the first 20 participants to determine if sample enrichment or adjustments to the protocol are required.

This will allow the estimation of the descriptive statistics of glucose concentration for the population (mean, standard deviation) at fasting, 60 minutes, and 120 minutes. Data from an adult study estimated the proportion of the population diagnosed as glucose intolerant as approximately 21% ^27^. If the proportion of the target population diagnosed as glucose intolerant was found to be 20%, and the sensitivity of the device was 100% then, with a sample size of 50, the 95% CI would be (69%, 100%) and if sensitivity were 90% the 95% CI would be (55%, 100%).

***6.3.2. Cohort 2***

We aim to recruit 45 children, to demonstrate acceptability and feasibility of the use of the capillary OGTT device at home. This number is justified based on the feasibility of being able to identify individuals known to be antibody-positive from established clinical or research platforms.

## 7. Data management

All study data will be entered on an electronic CRF (REDcap). Results from laboratory testing in central laboratory in the School of Medicine at Swansea University will be provided in batch, at the end of the study via an Excel spreadsheet using OneDrive (as per data transfer requirements set out by information compliance for confidential information). Results from local NHS laboratories will be entered onto REDCap by local research nurses.

All data will be processed according to the Data Protection Act 2018, and UK General Data Protection Regulation (GDPR), and all documents will be stored safely in confidential conditions. All study-specific documents will refer to the participant with a unique study participant number/code and not by name.

Participant identifiable data will be stored separately from study data and in accordance with University of Oxford Standard Operating Procedure 13 (confidentiality and security of personal data). All study documentation will be stored securely in offices only accessible by swipe card or key lock by the central coordinating team staff in Oxford and authorised personnel.

The University of Oxford, as per their requirements for paediatric studies, will keep essential documents for the time period of 3 years after the youngest subject reaches 18 years old or 5 years, whichever is longer. It will be stored electronically on the University of Oxford central server.

The local NHS Trust will retain study-related identifiable information as per NHS policy after the study has finished.

## 8. Safety considerations

To minimise the burden of the OGTT we will aim to recruit participants already scheduled for testing. The sensation of the lancet (finger pricker) device may be disconcerting for some children. To reduce discomfort, we will use a smaller lancet (1.5mm depth) for children under 8 years old. We will make it clear to the participants that the sampling can be stopped at any time, and that they are not obliged to complete it once begun.

In cohort 1, it is possible that the results differ between the capillary and venous blood test results and may cause concern to participants. The venous blood test results will remain the gold standard for patient care. As we are measuring haematocrit as part of a full blood count, there is a possibility of identifying abnormalities that were otherwise unknown. The local principal investigator will review these results, and if clinically significant, ensure that the test is repeated. In the event of a persistent abnormality, we will inform the family, GP or hospital doctor (where appropriate), and ensure the appropriate action is taken.

In cohort 2, it may be possible that we identify someone who is in stage 2 (dysglycaemia) or stage 3 (hyperglycaemia) and is not undergoing routine monitoring or treatment. The test results may therefore be beneficial in identifying individuals with clinical T1D, before they develop symptoms or life-threatening illness. In the situation of stage 2 T1D, we will inform and educate participants and their parents on the signs and symptoms of T1D and refer them back to their referring site (clinical care or research source) for further assessment. In the situation of stage 3 T1D, we will assess participants for signs and symptoms of T1D, refer them to their local hospital for further management, and inform their GP.

## 9. Dissemination plan

The outputs include publication to a peer-reviewed journal. We will present anonymised data at relevant regional, national and international conference(s). We will make the results available to our participants via a newsletter.

## 10. Discussion

There is considerable international momentum to screen children and young people for early-stage T1D, but questions remain on how best to monitor those at risk over time, during the latency period between autoimmunity and clinical disease. Studies have suggested there may be less invasive alternatives to the OGTT, including CGM. Although promising, evidence is mixed on the ability of CGM to match the diagnostic and prognostic performance of the OGTT ^36, 37^.

The OGTT remains an imperfect gold standard test, with significant variability in its reproducibility. It is also invasive and time-consuming, possibly contributing to marked attrition in follow-up studies ^15, 16^. Given it remains the gold standard for staging and assessing risk of progression, there is a clear need to improve upon some of these limitations. A capillary alternative, which measures glucose at the point of care using a small drop of blood, may help to overcome sample processing issues, avoiding glucose degradation over time whilst reducing invasiveness ^23– 25^.

This study aims to build on previous work done to assess the accuracy, feasibility and acceptability of the GTT@home device in adults ^27, 28^, by extending it to children and young people. Given the invasiveness of the standard OGTT, sample handling related issues, and the large proportion of children and young people lost to follow-up, a less invasive, but accurate alternative is needed for children, young people and their families.

## 11. Ethics and consent

This study has received ethical approval from the South Birmingham Research Ethics Committee (reference: 23/WM/0184, 21 ^st^ September 2023), is registered with the Health Regulation Authority, and will be conducted in accordance with the principles of the Declaration of Helsinki. All consent processes, including the consent forms have been approved by a Research Ethics Committee and are compliant with Good Clinical Practice, UK regulatory and legal requirements. Children with T1D (stages 1–3) and their families gave feedback on study materials before ethical approval. Informed consent will be obtained face-to-face for cohort 1, and remotely for cohort 2, as the study visit for this cohort will take place at their home. Investigators must ensure that study participants (or their legal guardian), are fully informed about the exact nature of the study, with any possible risks clearly outlined. Written informed consent with be obtained before any study-specific procedures are performed. Age-appropriate participant information sheets will be given to participants under 16 years of age, and they will be asked to sign an assent form, and parents will be asked to provide consent before the child can participate. Participants aged 16 years or above will be asked to consent as adults. If a participant has a second study visit (cohort 1) and turns 16 years old after their first visit, they will be re-consented before the second visit. Remote consenting will follow the same process, which will be completed by the study team via Microsoft Teams ^32^ and recorded as informed verbal consent (cohort 2).

## Data Availability

No data are associated with this article.
