# Supplementary material for: A study to determine a capillary alternative to the gold standard oral glucose tolerance test -  Protocol
Source: Wellcome Open Res. 2026 Jan 29;9:601. Originally published 2024 Oct 17. [Version 2] doi: 10.12688/wellcomeopenres.23028.2 (PMC11803194; doi:10.12688/wellcomeopenres.23028.2)
Supplement: Peer review history

# Reviewer report: Reviewer response for version 1

**DOI:** 10.21956/wellcomeopenres.25359.r112374

**Published:** 2024-12-17

**Authors:** David A Baidal

**Affiliations:** 1 Diabetes Research Institute, University of Miami, Coral Gables, Florida, USA

Swaby et al propose a protocol aimed at evaluating a capillary blood glucose testing alternative to the gold standard OGTT in subjects with early-stage Type 1 diabetes (Stage 1 and 2) and clinical (stage 3) type 1 diabetes. 

 The authors highlight the inconveniences/limitations associated with venous blood sampling during the conduction of an OGTT. They propose obtaining capillary blood glucose measurements, in lieu of venous blood samples, with the use of the GTT@home device which provide a blood glucose level from an enzymatic reaction using two glucose dehydrogenase sensors. 

 The protocol is of interest and attempts at simplifying the OGTT, particularly for children, are certainly needed.

**I have the following comments/questions:**

1. The authors state that the GTT@home device has a ULCA/CE mark to perform an OGTT at home. I would recommend providing a reference for this statement.
2. What is the minimum amount of blood required to test a capillary blood glucose level with the GTT@home device?
3. With regards to preparation for the OGTT, current guidelines require patients to follow a high carbohydrate diet of at least 150 g/day for 3 days prior to testing and this requirement should be added to the protocol.
4. What is the youngest age that will be included in the protocol? Children younger than 4 years may have difficulty in consuming the required high carbohydrate diet prior to the OGTT.
5. If the purpose of this protocol is to determine if the GTT@home device can be used in routine clinical care, why does the protocol include patients with clinical type 1 diabetes on insulin therapy? Monitoring “subjects at risk” with repeated OGTTs is a strategy to identify progression from Stage 1/Stage 2 to Stage 3 T1D.  Once Stage 3 T1D is diagnosed, there is no clinical indication to perform additional OGTTs. If there is an interest to evaluate beta cell function following clinical disease onset, then C-peptide measurements would be required, which is not the analyte being tested by the GTT@home device. Therefore, I am confused as to the rationale for including subjects with clinical T1D in Cohort 1. Please clarify.
6. There should be guidance in both cohort 1 (excluding patients with clinical T1D) and cohort 2 regarding the capillary blood glucose level at which the test should not be performed. In this context, a capillary blood glucose ≥11.1 mmol/L should be an indicator to contact the study team to determine if adequate to proceed with OGTT.

Is the study design appropriate for the research question?

Partly

Is the rationale for, and objectives of, the study clearly described?

Partly

Are sufficient details of the methods provided to allow replication by others?

Yes

Are the datasets clearly presented in a useable and accessible format?

Not applicable

Reviewer Expertise:

type 1 diabetes prevention and early intervention.

I confirm that I have read this submission and believe that I have an appropriate level of expertise to confirm that it is of an acceptable scientific standard, however I have significant reservations, as outlined above.

# Response

**Published:** 2026-01-22

**Authors:** Rabbi Swaby

**Affiliations:** Nuffield Department of Medicine, University of Oxford, Oxford, England, UK

We thank you for your helpful comments. We have responded to the best of our ability, below, with our responses after each comment, and adjusted the manuscript accordingly. As a result, we believe the manuscript is strengthened. We provide a clean and tracked new version of the manuscript, with any additional or changed wording underlined. **I have the following comments/questions:**

1. The authors state that the GTT@home device has a ULCA/CE mark to perform an OGTT at home. I would recommend providing a reference for this statement.

 
**RESPONSE**: Thank you for this suggestion. We have provided a reference to the UKCA/CE mark as suggested. **Reference**: Medicines and Healthcare products Regulatory Agency. Public access registration database. *Medicines and Healthcare products Regulatory Agency *2025. Date accessed: 23 July 2025. https://pard.mhra.gov.uk/manufacturer-details/44830.

  

1. What is the minimum amount of blood required to test a capillary blood glucose level with the GTT@home device?

 
**RESPONSE**: Please see full response to reviewer 2, minor comment 5. The device uses just 0.6 microlitres of capillary blood.  

1. With regards to preparation for the OGTT, current guidelines require patients to follow a high carbohydrate diet of at least 150 g/day for 3 days prior to testing and this requirement should be added to the protocol.

 
**RESPONSE**: Thank you for this suggestion. We agree that correct preparation is important for an OGTT. We request that participants continue a normal, balanced diet leading up to their OGTT. This is based on Public Health England guidance, which recommends that all children aged 4 years and above consume >150g/day of carbohydrate per day (except boys aged 2-3: ≥145g/day and girls aged 2-3 years: ≥134g/day). We have updated the manuscript to clarify this. **We have included these changes in the main text as follows**: **Study procedures, page 10, paragraph 6, lines 2-3, bullet point 1:** **Reference**: Public Health England. Government Dietary Recommendations: Government recommendations for energy and nutrients for males and females aged 1-18 years and 19+ years. *Public Health England* 2016. Date accessed: 21 July 2025. https://assets.publishing.service.gov.uk/government/uploads/system/uploads/attachment_data/file/618167/government_dietary_recommendations.pdf.  

1. What is the youngest age that will be included in the protocol? Children younger than 4 years may have difficulty in consuming the required high carbohydrate diet prior to the OGTT.

 
**RESPONSE**: This study invites children who have been assessed by a clinician or researcher, who have deemed it appropriate (and possible) to perform an OGTT in that individual. Given this, we have not set a lower age limit for study inclusion. As stated in response to comment 3, a normal balanced diet would contain >150g/day of carbohydrate, for children aged 4 years and above, and >145g/day in boys aged 2 years and above.

  

1. If the purpose of this protocol is to determine if the GTT@home device can be used in routine clinical care, why does the protocol include patients with clinical type 1 diabetes on insulin therapy? Monitoring “subjects at risk” with repeated OGTTs is a strategy to identify progression from Stage 1/Stage 2 to Stage 3 T1D.  Once Stage 3 T1D is diagnosed, there is no clinical indication to perform additional OGTTs. If there is an interest to evaluate beta cell function following clinical disease onset, then C-peptide measurements would be required, which is not the analyte being tested by the GTT@home device. Therefore, I am confused as to the rationale for including subjects with clinical T1D in Cohort 1. Please clarify.

 
**RESPONSE**: Thank you for this question. This study aims to assess if the capillary OGTT device shows good agreement and diagnostic accuracy compared with venous glucose measures across the standard diagnostic glucose thresholds, including the threshold for the diagnosis of T1D (11.1 mmol/L). For this, glucose values across the whole diagnostic range are needed. Therefore, children with known T1D are invited to ensure we are able to assess the capillary device when glucose values are above 11.1 mmol/L.  

  

1. There should be guidance in both cohort 1 (excluding patients with clinical T1D) and cohort 2 regarding the capillary blood glucose level at which the test should not be performed. In this context, a capillary blood glucose ≥11.1 mmol/L should be an indicator to contact the study team to determine if adequate to proceed with OGTT.

 
**RESPONSE**: This protocol was designed after review of OGTT protocols used in clinical practice and research studies across the UK. It would not align with current clinical practice in the UK to rely on a capillary glucose to determine eligibility for an OGTT in the assessment for T1D/T2D. Given they are insulin-deficient, participants with known stage 3 T1D have their glucose checked before their OGTT, to ensure it is safe to proceed ( **see Study Procedures, page 10/11**). In cohort 2, our target population does not include children with stage 3 T1D, so participants are assessed against exclusion criteria before study entry, which includes no evidence of overt stage 3 T1D ( **see page 9, Table 5, exclusion criteria, cohort 2**).   To clarify, the GTT@home device does not function like a standard glucometer. It does not have a screen and requires scanning after the full OGTT is complete to transfer the glucose data to a secure database for study team review. For safety, participants in cohort 1 are managed by the team performing the OGTT, and the clinical venous glucose samples taken are used to determine OGTT outcome. For cohort 2, the study team review the capillary glucose levels same day (or next working day if at a weekend) and inform the referring team (and local diabetes team), if the threshold for stage 3 T1D is met ( **see Safety Considerations, page 15, paragraph 2**). In this situation, the study team would also review the participant (remotely) to ensure they remain well and are showing no immediate signs of diabetes.

# Reviewer report: Reviewer response for version 1

**DOI:** 10.21956/wellcomeopenres.25359.r110325

**Published:** 2024-12-05

**Authors:** Christopher J. Nolan

**Affiliations:** 1 Australian National University, Canberra, Australian Capital Territory, Australia

Predicting stage 3 of T1D in children at risk through repeated glucose tolerance testing is valuable, as it can reduce the risk of first presentation of Stage 3 in diabetic ketoacidosis. To reduce the burden of this test on children and young people through an at home test would be an advance. Would be great for those living in rural and remote sites in particular.

 This study with 2 cohorts - first to assess the performance of the capillary alternative OGTT method against the gold standard test with venous sampling - second to road test the capillary alternative with participants at risk of T1D at home. This is an appropriate design. I have just a few major comments that the research team should consider.

**Major comments:**

 1) The goal is to have a test suitable for children and young people. It would be helpful to state recruitment targets for age group brackets. Also is there an age limit below which children will be excluded?

 2) It is unclear as to why participants with established T1D will be recruited in Cohort 1, giving them an oral glucose load without bolus insulin even if their fasting glucose is up to 15 mmol/L. If stage 3 T1D participants are to be included, suggest allow an insulin bolus (perhaps at 50% of what would be predicted to be required according to their insulin to carbohydrate ratio).

 3) Were young people at risk of T1D and parents involved in the study design, if so this needs to be stated?

 4) The post-test questionnaires will be valuable. However, it would also be worth considering post-test interviews with the young people and their parents, either individually or in groups, using qualitative research methods. Elements of their experience with the home test may be missed through questionnaires only.

 5) How will the 2 ml NaF venous sample be processed? On ice? Time allowed pre-centrifugation?

**Minor comments:**

 1) Table 1 - for stage 2 - should say 'Dysglycaemia' - as it is more than just 'impaired fasting glucose'.

 2) Table 2 - suggest add to second column heading - Number of **OGTT** timepoints

 3) Table 3- for CGM metrics - is it **mean** glucose >7.8 mmol/l or another metric around 7.8 mmol/L (e.g. a percentage of time above 7.8 mM). a mean >7.8 mmol/L seems very high??

 4) In text section 2.2 - Instead of "Stages 1-2 precede clinical disease (stage 3), requiring insulin treatment" suggest "Stages 1-2 precede clinical disease requiring insulin treatment (stage 3)".

 5) What is the volume of blood (microL) required for the capillary test?

Is the study design appropriate for the research question?

Yes

Is the rationale for, and objectives of, the study clearly described?

Yes

Are sufficient details of the methods provided to allow replication by others?

Yes

Are the datasets clearly presented in a useable and accessible format?

Not applicable

Reviewer Expertise:

Senior clinician scientist working in the field of diabetes.

I confirm that I have read this submission and believe that I have an appropriate level of expertise to confirm that it is of an acceptable scientific standard.

# Response

**Published:** 2026-01-22

**Authors:** Rabbi Swaby

**Affiliations:** Nuffield Department of Medicine, University of Oxford, Oxford, England, UK

We thank you for your helpful comments. We have responded to the best of our ability, below, with our responses after each comment, and adjusted the manuscript accordingly. As a result, we believe the manuscript is strengthened. We provide a clean and tracked new version of the manuscript, with any additional or changed wording underlined. **Major comments:**

 1) The goal is to have a test suitable for children and young people. It would be helpful to state recruitment targets for age group brackets. Also is there an age limit below which children will be excluded?  

**RESPONSE**: We did not create recruitment targets based on age, but instead based on glycaemic status, as our main aim is to assess the capillary device across the whole diagnostic range ( **see** **page 5, paragraph 11, lines 2-4**). We did not set a lower age limit for this study. This study aims to assess the capillary device across the childhood age range, and so we wanted to allow younger participants to take part, if it was deemed appropriate by their referring team. This study invites children who have been deemed appropriate to attempt an OGTT, as assessed by either their clinical or research team. If they agree to take part, their research samples are in addition to the ones already being taken. We feel it is important to assess the capillary OGTT device in the youngest age groups, as they are the ones who stand to benefit most, in terms of test invasiveness and accessibility.

 2) It is unclear as to why participants with established T1D will be recruited in Cohort 1, giving them an oral glucose load without bolus insulin even if their fasting glucose is up to 15 mmol/L. If stage 3 T1D participants are to be included, suggest allow an insulin bolus (perhaps at 50% of what would be predicted to be required according to their insulin to carbohydrate ratio).  

**RESPONSE**: To test the capillary OGTT device across the diagnostic glycaemic range, a spread of glucose values, ranging from normoglycaemia to hyperglycaemia ( **as per Table 1, page 3**) is needed. For this reason, children with known T1D were invited to take part and are monitored post-test to ensure they remain well, with prompt correction of their glucose levels. We agree that the glucose load was too high for children with stage 3 T1D, so we amended the protocol to reduce the glucose load to 1g/kg oral glucose. When checking the participants glucose prior to the test, we have reduced the upper limit to continue from 15 mmol/L to 11 mmol/L **We have included these changes in the main text as follows**: **Study procedures, page 11, bullet point 9**: The glucose drink will be consumed within 10 minutes (1.75g/kg or 75g max). For participants with known stage 3 T1D, reduce oral glucose dose (1g/kg or max 75g). **Study procedures, page 11, bullet point 4**: Those with T1D will continue their long-acting insulin, omit their morning rapid-acting insulin and have their blood glucose checked. If the result is 4 – 11mmol/L they can proceed. If their blood glucose is >15 mmol/L they must check their ketones and treat appropriately, and not proceed.

 3) Were young people at risk of T1D and parents involved in the study design, if so this needs to be stated?  

**RESPONSE**: Children with T1D (stages 1-3) were involved in development of study materials. We have updated the manuscript to include this detail. **We have included these changes in the main text as follows**: **Ethics and consent, page 16,** **paragraph 3, lines 6-7**: Children with T1D (stages 1-3) and their families gave feedback on study materials before ethical approval.

 4) The post-test questionnaires will be valuable. However, it would also be worth considering post-test interviews with the young people and their parents, either individually or in groups, using qualitative research methods. Elements of their experience with the home test may be missed through questionnaires only.  

**RESPONSE**: Yes, the authors agree that qualitative interviews would capture valuable data often missed using questionnaires. We are currently undertaking a qualitative study to better understand the views and experiences of young people, parents and healthcare professionals involved in metabolic monitoring, and how this can be integrated in routine clinical care.

 5) How will the 2 ml NaF venous sample be processed? On ice? Time allowed pre-centrifugation?  

**RESPONSE**: The 2ml venous fluoride oxalate glucose samples are processed within 30 minutes. The samples are taken immediately to the laboratory to undergo centrifugation, before aliquoting 1ml of plasma, which is frozen at -20 to -80˚C. We have updated the manuscript with this detail. **We have included these changes in the main text as follows**: **Study procedures, page 11, lines 20-21, bullet point 15**: All venous glucose samples will be processed (centrifugation, freezing ≥1ml of plasma [-20 to -80˚C] within 30 minutes).

**Minor comments:**

 1) Table 1 - for stage 2 - should say 'Dysglycaemia' - as it is more than just 'impaired fasting glucose'.  

**RESPONSE**: Thank you for this suggestion. The word dysglycaemia has been added to **Table 1**, **column 3**, **row 3** on **page 3**. We have also updated **column 3, row 2** to use the word normoglycaemia, as this section also reflects more than just fasting glucose.

 2) Table 2 - suggest add to second column heading - Number of **OGTT **timepoints

**RESPONSE**: Thank you for this suggestion, we have updated **Table 2** to add the word “ **OGTT**” to **column 2**, **row 1**, on **page 4**.

 3) Table 3 - for CGM metrics - is it **mean** glucose >7.8 mmol/l or another metric around 7.8 mmol/L (e.g. a percentage of time above 7.8 mM). a mean >7.8 mmol/L seems very high??  

**RESPONSE**: The CGM metric stated in Table 3 refers to the percentage time spent above 7.8 mmol/L. We have updated the sentence to clarify this.

**We have included these changes in the main text as follows**: **Table 3, page 5, column 4, row 4: **Risk of progression to stage 3 T1D over 1 year: 10% time >7.8mmol/L (140mg/dL) and over 2 years: 5% time >7.8mmol/L.

   4) In text section 2.2 - Instead of "Stages 1-2 precede clinical disease (stage 3), requiring insulin treatment" suggest "Stages 1-2 precede clinical disease requiring insulin treatment (stage 3)".  

**RESPONSE**: Thank you for this. We have updated the sentence with your suggested rephrasing.

 5) What is the volume of blood (microL) required for the capillary test?  

**RESPONSE**: The capillary test device requires 0.6 microL of capillary blood. We have updated the manuscript to clarify this important point. **We have included these changes in the main text as follows**: **Table 3, page 6, paragraph 2, lines 3-4: **It is calibrated to convert capillary to venous plasma glucose results, using 0.6 microliters of capillary blood.

# Reviewer report: Reviewer response for version 1

**DOI:** 10.21956/wellcomeopenres.25359.r108394

**Published:** 2024-11-20

**Authors:** Daria Di Filippo

**Affiliations:** 1 University of New South Wales, Sydney, New South Wales, Australia

This study aims to assess the accuracy, feasibility, and acceptability of a capillary alternative to the gold standard OGTT to be performed at home in monitoring the evolution towards clinical T1DM in children.

 The rationale, objectives, study design, methods and dataset planning are presented clearly and in-depth, allowing for replication by others and appearing well-planned.

 I support this study as potentially creating a more acceptable test for children and their families, overcoming the many limitations of the OGTT while awaiting the development of more advanced diagnostic techniques (as the validation of CGM).

 I look forward to read the results of this study in confirming the valid research points outlined above.

Is the study design appropriate for the research question?

Yes

Is the rationale for, and objectives of, the study clearly described?

Yes

Are sufficient details of the methods provided to allow replication by others?

Yes

Are the datasets clearly presented in a useable and accessible format?

No

Reviewer Expertise:

In my PhD I assessed CGM for the diagnosis of Gestational Diabetes. Being aware of the numerous limitations of the OGTT, I have an interest in research looking at overcoming these improving acceptability for patients.

I confirm that I have read this submission and believe that I have an appropriate level of expertise to confirm that it is of an acceptable scientific standard, however I have significant reservations, as outlined above.
